# Can the frailty score independently predict postoperative morbidity in patients with colorectal cancer? A prospective observational study

**DOI:** 10.1186/s12877-026-07255-7

**Published:** 2026-02-27

**Authors:** Mustafa Kemal Sahin, Belkis Yilmaz, Arif Timuroglu

**Affiliations:** Department of Anesthesiology, Dr. Abdurrahman Yurtaslan Oncology Training and Research Hospital, Ankara, Turkey

**Keywords:** Frailty, Colorectal Neoplasms, Postoperative Complications, Geriatric Assessment, Mortality

## Abstract

**Background:**

Frailty is associated with adverse surgical outcomes in older adults. We evaluated whether the Edmonton Frail Scale (EFS) is independently associated with 30-day postoperative morbidity and mortality after major colorectal cancer surgery.

**Methods:**

This prospective observational study enrolled patients aged ≥ 65 years undergoing elective oncologic colorectal resection (March–September 2025). Patients were stratified into five EFS frailty categories. The primary outcomes were 30-day postoperative complications (Clavien–Dindo; Grade I–II vs Grade III–V) and 30-day all-cause mortality. Associations were assessed using ROC analysis and multivariable logistic regression.

**Results:**

Of 205 enrolled patients, 200 were analyzed (63% male; median age 70 years). Thirty-day postoperative complications occurred in 40 patients (20%), and 30-day mortality was 5% (10/200). EFS was associated with longer hospital length of stay and higher complication and mortality rates. EFS showed excellent discrimination for postoperative complications (AUC 0.928; 95% CI 0.886–0.970), with an optimal cut-off of ~ 6.5 (sensitivity 96.2%, specificity 76.9%). In multivariable models, EFS remained independently associated with complications (OR 1.284; *p* = 0.006) and mortality (OR 1.323; *p* = 0.014).

**Conclusions:**

Preoperative EFS provides independent and clinically meaningful prediction of 30-day morbidity and mortality after major colorectal cancer surgery in older adults and may enhance perioperative risk stratification and shared decision-making.

**Trial registration:**

NCT06866678 and registration on 05 March 2025.

## Introduction

Frailty is a multidimensional geriatric syndrome characterized by reduced physiological reserve and increased vulnerability to stressors in older adults. It is associated with adverse outcomes such as falls, disability, hospitalization, and mortality [1, 2] and its prevalence increases with age, affecting approximately 17% of community-dwelling older adults and more than half of those aged > 90 years [3]. Frailty is not an inevitable consequence of aging; rather, it reflects cumulative dysregulation across multiple organ systems, including musculoskeletal, endocrine, immune, and cardiovascular pathways [4].

Multiple frailty instruments have been developed, including the physical frailty phenotype, deficit-accumulation indices, and multidimensional tools [5]. The Edmonton Frail Scale (EFS) is a validated and practical instrument that enables rapid preoperative assessment in routine clinical settings [6]. Frailty assessment is particularly relevant in patients aged ≥ 65 years undergoing major surgery, as frailty has been consistently associated with higher postoperative complication rates, prolonged hospitalization, and increased mortality [7, 8]. Incorporating multidimensional frailty screening into perioperative care may therefore improve risk communication, perioperative planning, and shared decision-making [9]. The literature consistently demonstrates that elderly patients with higher levels of frailty experience significantly increased rates of postoperative complications and longer hospitalizations [7]. Thus, systematic assessment using the EFS is an indispensable tool for identifying perioperative risks and enhancing patient safety in the surgical setting.

At SBU Dr. Abdurrahman Yurtaslan Oncology Training and Research Hospital, major colorectal cancer surgeries are frequently performed, and a significant proportion of this patient group consists of individuals over the age of 65. The elderly cancer population is considerably more vulnerable and frailer compared to the general population, due to both the underlying malignancy and age-related physiological changes [10]. Therefore, objective assessment of frailty using validated scales in the preoperative period enables clinicians to better predict critical postoperative outcomes such as complications, mortality, and length of hospital stay. The use of comprehensive and practical tools like the EFS facilitates multidimensional evaluation of patients and supports more accurate preoperative risk stratification [3]. Although the literature on the impact of frailty scales on postoperative outcomes in elderly patients undergoing cancer surgery is limited, recent studies have demonstrated that higher EFS scores are significantly associated with increased postoperative complications, mortality, and prolonged hospitalization in this population [5, 7]. Thus, systematic frailty assessment with the EFS is an essential component of perioperative care for older adults with cancer.

The aim of this study is to investigate the relationship between preoperative frailty assessment using the EFS and postoperative outcomes such as complications, mortality, and length of hospital stay in patients over 65 years of age undergoing major colorectal cancer surgery at our institution. By addressing this topic, our study seeks to fill a significant gap in the current literature, as there are limited data on the prognostic value of frailty assessment tools like the EFS in elderly cancer surgery patients.

## Methods

This prospective observational study was conducted at Dr. Abdurrahman Yurtaslan Oncology Training and Research Hospital between March 2025 and September 2025. Ethical approval was obtained from the Dr. Abdurrahman Yurtaslan Oncology Training and Research Hospital Clinical Research Ethics Committee (Approval No: 2025–02/28). The study was conducted in accordance with the Declaration of Helsinki, registered at ClinicalTrials.gov (NCT06866678; 05 March 2025), and reported in line with the STROBE guidelines.

Frailty was assessed preoperatively using the Edmonton Frail Scale (EFS), a brief and validated multidimensional instrument covering nine domains: cognition, general health, functional independence, social support, medication use, nutrition, mood, urinary continence, and functional performance. The EFS was administered before surgery by trained clinicians from the anesthesiology team. Based on the total score, patients were categorized into five predefined groups: non-frail (0–5), vulnerable [6, 7], mildly frail [8, 9], moderately frail [10, 11], and severely frail [12–17]. For each participant, we recorded demographic characteristics (age, sex, body mass index), perioperative risk status (ASA physical status), and key intraoperative variables (operative duration and estimated blood loss). Tumor stage (TNM stage) and neoadjuvant treatment (chemotherapy, radiotherapy, or combined chemoradiotherapy) were also documented [11].

Eligible patients were adults scheduled for elective oncologic colorectal resection for colorectal cancer. Consecutive eligible patients during the study period were approached for inclusion, and those who consented were enrolled. Operations consisted of standard oncologic colorectal resections (including hemicolectomy, subtotal/total colectomy, low anterior resection, and abdominoperineal resection), with or without stoma formation, according to routine practice at our institution. The operative approach was recorded as open or laparoscopic. When a laparoscopic procedure required conversion to an open operation, the case was analyzed within the open surgery category. More granular procedural details—such as tumor location (colon vs rectum), extent of lymphadenectomy, specific dissection planes, and anastomotic technique—were not captured in a standardized format in the case report forms and therefore were not included in procedure-level analyses.

The primary outcomes were 30-day postoperative complications (medical and surgical) and 30-day all-cause mortality. Complications occurring during the index hospitalization were recorded prospectively using predefined clinical criteria. To capture post-discharge events, patients (or their caregivers) were contacted by telephone and asked about complications, hospital readmissions, re-interventions, and death within 30 days after surgery; this information was incorporated into the final 30-day dataset. Complication severity was graded using the Clavien–Dindo classification. For the main analyses, complications were evaluated both as overall occurrence (yes/no) and by severity (Grade I–II vs Grade III–V; Grade V indicates death). Although Grade V corresponds to death in the Clavien–Dindo system, mortality was also analyzed separately as a distinct endpoint. Pulmonary complications were defined according to European Perioperative Clinical Outcome (EPCO) criteria [12]. Other medical complications (e.g., sepsis, cardiac, neurological, and renal events) were defined using standard ACS NSQIP definitions, and delirium was diagnosed clinically by the treating team in accordance with DSM-5 criteria [13]. Surgical complications (e.g., anastomotic leakage, surgical site infection, postoperative ileus, postoperative hemorrhage, and evisceration) were recorded from clinical documentation and graded within the Clavien–Dindo framework. Postoperative outcomes were recorded prospectively using predefined clinical criteria and entered into the study database by the research team. Secondary outcomes were PACU length of stay and total hospital length of stay. Unless otherwise specified, the term ‘postoperative complications’ refers to both medical and surgical events occurring within 30 days after surgery.

Analyses were performed using SPSS (version 25; IBM Corp., Armonk, NY, USA). We did not apply the ACS NSQIP Surgical Risk Calculator because the dataset did not include the full set of required NSQIP-specific preoperative variables and procedure-level coding. Continuous variables are presented as mean ± standard deviation or median (interquartile range), and categorical variables as counts and percentages. Comparisons across frailty categories were conducted using Chi-square or Fisher’s exact tests for categorical variables and one-way ANOVA or Kruskal–Wallis tests for continuous variables, as appropriate. Correlations between EFS and outcomes were assessed with Spearman’s rho. Discrimination was examined using receiver operating characteristic (ROC) analysis, with the optimal cut-off defined by Youden’s index. Univariable and multivariable logistic regression models were used to identify independent predictors of postoperative complications and mortality; variables considered clinically relevant and/or associated with outcomes in univariable analyses were entered into the multivariable models. Model calibration was assessed with the Hosmer–Lemeshow test, and discrimination was summarized using the area under the ROC curve (AUC). A two-sided p-value < 0.05 was considered statistically significant.

## Results

A total of 205 patients were enrolled. Five patients were excluded because of missing or erroneous data, leaving 200 patients for the final analysis. According to preoperative EFS scores, patients were classified into five frailty categories: non-frail (*n* = 28), vulnerable (*n* = 49), mildly frail (*n* = 54), moderately frail (*n* = 38), and severely frail (*n* = 31). Baseline demographic, clinical, and perioperative characteristics across frailty groups are summarized in Table 1.

**Table 1:** Demographic data

		**Total** *n* = 200	**Non frail** *n* = 28	**Vulnerable** *n* = 49	**Mildly Frail** *n* = 54	**Moderate Frailty** *n* = 38	**Severe Frailty** *n* = 31	p
Gender, n (%)								0.183*
Man		126 (63)	23 (82)	28 (57)	33 (61)	25 (66)	17 (55)	
Woman		74 (37)	5 (18)	21 (43)	21 (39)	13 (34)	14 (45)	
Age, median (IQR)			70 (65–73)	70 (65–79)	74 (68–82)	78 (71–86)	77 (72–89)	**0.028**^**+**^
BMI, mean (SD)			24 ± 0.69	25 ± 0.70	25 ± 0.44	26 ± 0.48	25 ± 0.76	0.336^−^
ASA, n (%)								0.000*
1		1 (1)	0 (0)	1 (2)	0 (0)	0 (0)	0 (0)	
2		79 (40)	15 (54)	26 (53)	27 (50)	8 (21)	3 (10)	
3		109 (54)	13 (46)	22 (45)	27 (50)	28 (74)	19 (61)	
4		11 (5)	0 (0)	0 (0)	0 (0)	2 (5)	9 (29)	
Surgical duration min, mean (SD)			229 ± 21	188 ± 8	183 ± 9	179 ± 10	213 ± 14	**0.027**^**−**^
Amount of bleeding, ml, mean (SD)			300 ± 57	209 ± 26	285 ± 60	217 ± 31	439 ± 82	**0.036**^**−**^
Type of disease, n (%) TNM	Stage 0	14 (7)	6 (21)	3 (6)	3 (6)	2 (5)	0 (0)	0.001*
	Stage 1	46 (23)	7 (25)	18 (37)	15 (28)	4 (11)	2 (6)	
	Stage 2	64 (32)	10 (36)	16 (33)	20 (37)	12 (32)	6 (19)	
	Stage 3	52 (26)	4 (14)	7 (14)	12 (22)	15 (39)	14 (46)	
	Stage 4	24 (12)	1 (4)	5 (10)	4 (7)	5 (13)	9 (29)	
Type of Surgery, n (%)	Open	46 (23)	6 (21)	11 (22)	12 (22)	9 (24)	8 (26)	0.882*
	Laparoscopic	154 (67)	22 (79)	38 (78)	42 (78)	29 (76)	23 (74)	
Neoadjuvant therapy, n (%)	Chemotherapy	9	1	2	3	2	1	0.785^‡^
	Radiotherapy	5	0	1	1	3	0	
	Chemotherapy and radiotherapy	12	3	3	1	2	3	

Data are presented as n (%), mean ± standard deviation, or median (interquartile range) as appropriate. *P*-value calculated by *Chi-square test

Bold values indicate statistical significance (*p* < 0.05)

^+^Kruskal-Walli’s test;—One-way ANOVA

^‡^Fisher’s Exact Test

Frailty status was examined in relation to postoperative recovery, specifically PACU length of stay and total hospital length of stay, by comparing non-frail patients (*n* = 28) with frail patients (*n* = 172). Frailty was not associated with a longer PACU stay, whereas frail patients experienced a significantly prolonged hospital length of stay. These findings are detailed in Table 2.

**Table 2:** The relationship between frailty and the length of stay in the postoperative care unit and hospital

	**Non frail** (*n* = 28)	**Frail** (*n* = 172)	p
PACU (Post-Anesthesia Care Unit), hours (mean ± SD)	24.7 ± 18,7	28.3 ± 17,6	0.312*
LOS (Length of Hospital Stay), days (mean ± SD)	7.1 ± 3	8.8 ± 5,6	0.023*

Bold values indicate statistical significance (*p* < 0.05)

^*^Independent Samples T-test

Associations between EFS scores and clinical outcomes (hospital length of stay, PACU length of stay, postoperative complications, and mortality) were assessed using Spearman’s rank correlation. EFS showed a statistically significant positive correlation with hospital length of stay (r = 0.222, *p* = 0.002). No significant correlation was observed between EFS and PACU length of stay (r = 0.117, *p* = 0.099). Higher EFS scores were also positively correlated with the occurrence of postoperative complications (r = 0.568, *p* = 0.026) and with mortality (r = 0.158, *p* = 0.025). These results are summarized in Table 3.

**Table 3:** Correlation between EFS and length of stay, PACU duration, postoperative complications, and mortality

	**Correlation Coefficient (r)**	**p value**
Edmonton Score – LOS (days)	0.222	0.002*
Edmonton Score – PACU (hours)	0.117	0.099*
Edmonton Score – Postoperative Complications	0.568	0.026*
Edmonton Score – Mortality	0.403	0.001*

*LOS *length of hospital stay, *PACU *Post-Anesthesia Care Unit, *EFS *Edmonton frail scale

Bold values indicate statistical significance (*p* < 0.05)

^*^Spearman’s rho

The association between frailty status and 30-day postoperative outcomes was evaluated (Table 4). Overall, 40 patients (20%) experienced at least one postoperative medical complication within 30 days, and complication rates differed significantly across frailty groups (*p* < 0.001). The proportion of patients with complications was 18% in the non-frail group (5/28), 18% in the vulnerable group (9/49), 17% in the mildly frail group (9/54), 8% in the moderately frail group (3/38), and 45% in the severely frail group (14/31). Thirty-day all-cause mortality was 5% overall (10/200) and also varied significantly by frailty status (*p* < 0.001), with mortality rates of 4% (1/28) in non-frail, 0% (0/49) in vulnerable, 4% (2/54) in mildly frail, 0% (0/38) in moderately frail, and 23% (7/31) in severely frail patients.

**Table 4:** Association between EFS and 30-day postoperative medical complications, Clavien–Dindo classification, and mortality

		Non Frail (*n* = 28)	Vulnerable (*n* = 49)	Mildly Frail (*n* = 54)	Moderate Frailty (*n* = 38)	Severe Frailty (*n* = 31)	Total (*N* = 200)	p
Postoperative Medical Complications, n (%)	No	23 (82)	40 (82)	45 (83)	35 (92)	17 (55)	160 (80)	< 0.001*
	Yes	5 (18)	9 (18)	9 (17)	3 (8)	14 (45)	40 (20)	
Clavien Dindo grade distribution, n	Grade 1	2	2	3	2	2	11	**0.003**^**+**^
	Grade 2	3	4	6	3	3	19	
	Grade 3	1	2	2	2	4	11	
	Grade 4	0	1	1	1	5	8	
Mortality, n (%)	Yes	1 (4)	0 (0)	2 (4)	0 (0)	7 (23)	10 (5)	** < 0.001**^**+**^
	No	27 (96)	49 (100)	52 (96)	38 (100)	24 (77)	190 (95)	

*P*-value calculated, *Chi-square test, + Fisher’s Exact Test. Categorical variables are presented as n (%). 'Postoperative medical complications' refers specifically to non-surgical systemic events (e.g., pulmonary, cardiac, delirium). For Clavien–Dindo classification, each patient was assigned a single grade corresponding to the highest severity observed within 30 days (patient-level highest grade), encompassing both medical and surgical complications. Mortality represents Clavien–Dindo Grade 5

*EFS *Edmonton frail scale

Bold values indicate statistical significance (*p* < 0.05)

The distribution of specific postoperative complications within 30 days, stratified by frailty status, is shown in Table 5. Pulmonary complications were the most frequent event (18 events overall), followed by surgical site infection (11 events) and postoperative ileus (9 events). Sepsis occurred in 7 events, and anastomotic leakage was recorded in 4 events. Cardiac and neurological complications were observed in 5 and 3 events, respectively; delirium occurred in 4 events, and renal replacement therapy was required in 3 events. Overall, 69 complication events were recorded across the cohort within 30 days (Table 5).

**Table 5:** Distribution of postoperative complications within the first 30 days according to frailty status

Complication	Total (*N* = 200)	Non Frail (*n* = 28)	Vulnerable (*n* = 49)	Mildly Frail (*n* = 54)	Moderate Frailty (*n* = 38)	Severe Frailty (*n* = 31)
Sepsis	7	0	2	3	0	2
Pulmonary complications	18	4	2	4	1	7
Cardiac complications	5	1	2	0	1	1
Neurological complications	3	0	1	1	1	0
Delirium	4	0	1	0	0	3
Renal replacement therapy	3	0	1	1	0	1
Anastomotic leakage	4	0	2	0	1	1
Surgical site infection	11	3	1	4	1	2
Postoperative ileus	9	1	0	2	2	4
Postoperative hemorrhage	3	0	0	1	0	2
Evisceration	2	0	0	1	1	0
Total number of complications (events)	69	9	12	17	8	23

Data are presented as absolute counts (number of events). More than one complication could occur in the same patient; therefore, the total number of events may exceed the number of patients with ≥ 1 complication

**Fig. 1:**
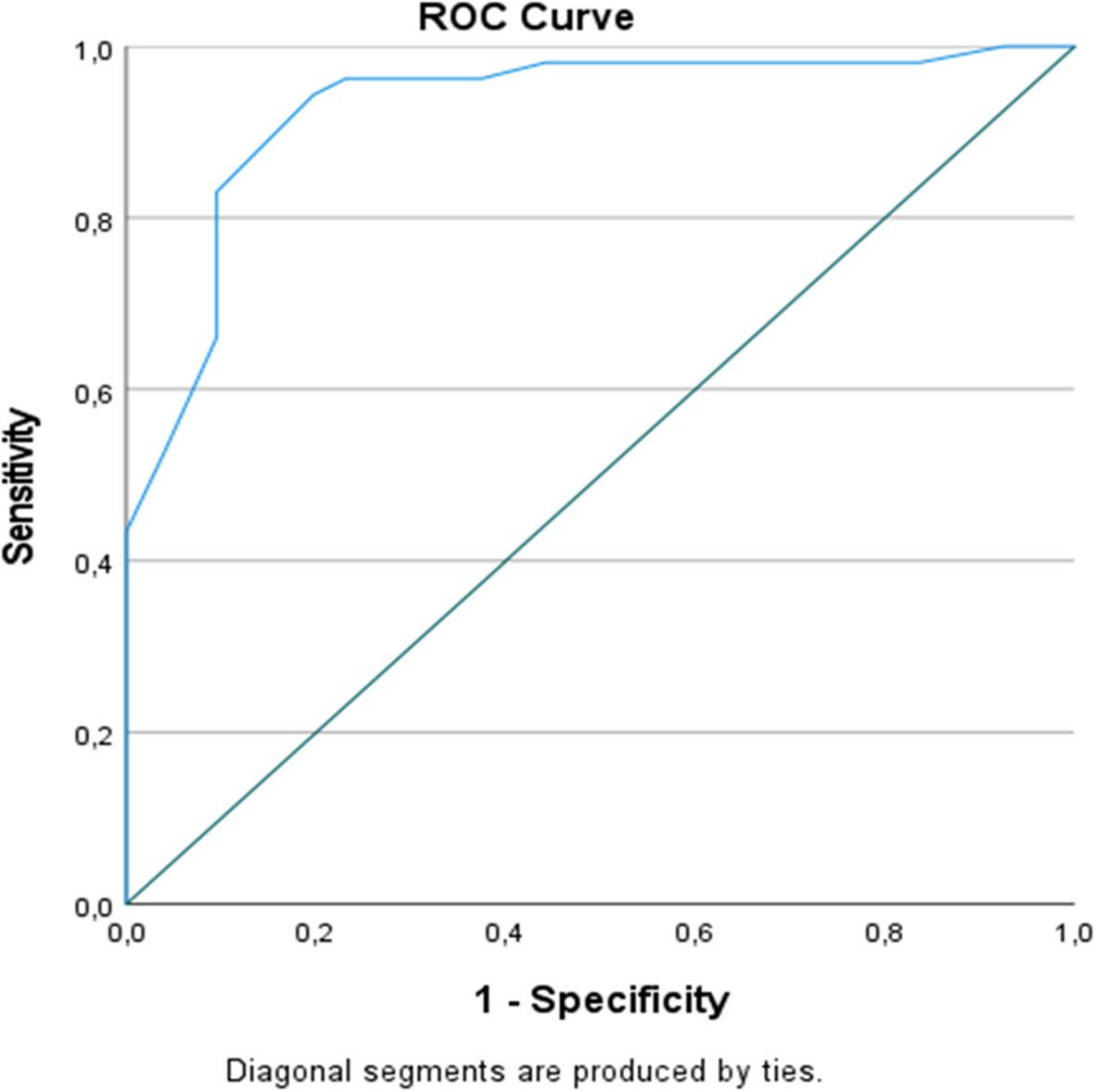
ROC curve demonstrating the predictive value of the Edmonton Frail Scale for postoperative complications

To evaluate the association between the Edmonton Frail Scale (EFS) and the development of postoperative complications, a receiver operating characteristic (ROC) analysis was performed (Fig. 1). The analysis demonstrated that the EFS had excellent discriminative ability for predicting postoperative complications, with an area under the curve (AUC) of 0.928 (95% CI: 0.886–0.970, *p* < 0.001). Based on the ROC curve analysis, an EFS cut-off value of approximately 6.5 provided the most favorable balance between sensitivity and specificity. At this threshold, the sensitivity was 96.2%, and the specificity was 76.9%, indicating a high ability of the EFS to correctly identify patients at risk for postoperative complications while maintaining acceptable specificity. These findings suggest that the EFS is a robust and reliable preoperative risk stratification tool for predicting the development of postoperative complications.

Univariable and multivariable logistic regression analyses of factors associated with 30-day postoperative complications are presented in Table 6. In univariable analysis, higher EFS score was associated with increased odds of postoperative complications (B = 0.290, *p* = 0.001; OR = 1.336, 95% CI: 1.125–1.586). Higher ASA physical status was also associated with complications (overall *p* < 0.001), with significant increases observed for ASA II (OR = 2.054, 95% CI: 1.020–4.130; *p* = 0.045), ASA III (OR = 3.670, 95% CI: 1.460–9.210; *p* = 0.006), and ASA IV (OR = 7.030, 95% CI: 2.260–21.900; *p* = 0.001) compared with ASA I. Longer operative duration (per 10 min) was associated with complications (B = 0.085, *p* = 0.004; OR = 1.089, 95% CI: 1.028–1.153). Advanced tumor stage (III–IV vs 0–II) (B = 0.480, *p* = 0.030; OR = 1.616, 95% CI: 1.050–2.485) and open surgical approach (vs laparoscopic) (B = 0.620, *p* = 0.012; OR = 1.859, 95% CI: 1.140–3.030) were also significant in univariable models.

**Table 6:** Univariate and multivariate analyses of risk factors for 30-day postoperative complications

Variable	Univariate B	p-value	Exp(B)	95% CI for Exp(B)	Multivariate B	p-value	Exp(B)	95% CI for Exp(B)
Edmonton score	0.290	0.001	1.336	1.125–1.586	0.250	**0.006**	1.284	1.075–1.535
Age (years)	0.020	0.190	1.020	0.990–1.051	0.010	0.560	1.010	0.977–1.044
Gender (male)	0.140	0.510	1.150	0.770–1.720	0.070	0.760	1.073	0.710–1.620
BMI	−0.022	0.650	0.978	0.895–1.069	−0.012	0.810	0.988	0.900–1.086
ASA (ref: I)		< 0.001				**0.004**		
ASA II	0.720	0.045	2.054	1.020–4.130	0.600	0.080	1.822	0.930–3.570
ASA III	1.300	0.006	3.670	1.460–9.210	1.120	**0.014**	3.065	1.260–7.460
ASA IV	1.950	0.001	7.030	2.260–21.900	1.700	**0.003**	5.470	1.800–16.650
Surgery duration (per 10 min)	0.085	0.004	1.089	1.028–1.153	0.070	**0.012**	1.073	1.015–1.134
Tumor stage (III–IV vs 0–II)	0.480	0.030	1.616	1.050–2.485	0.390	**0.047**	1.477	1.006–2.170
Surgical approach (Open vs Lap [ref])	0.620	0.012	1.859	1.140–3.030	0.520	**0.028**	1.682	1.060–2.670
Neoadjuvant treatment (Any vs None [ref])	0.160	0.520	1.174	0.720–1.920	0.080	0.740	1.083	0.680–1.720

Model fit: Hosmer–Lemeshow test: χ^2^ = 5.84, *p* = 0.665; AUC (95% CI): 0.76 (0.70–0.82)

*Abbreviations*: *CI* confidence interval, *BMI* body mass index, *ASA *American Society of Anesthesiologists physical status

Bold values indicate statistical significance (*p* < 0.05)

In the multivariable model, EFS remained independently associated with 30-day postoperative complications (B = 0.250, *p* = 0.006; OR = 1.284, 95% CI: 1.075–1.535). ASA physical status remained significant overall (*p* = 0.004), with ASA III (OR = 3.065, 95% CI: 1.260–7.460; *p* = 0.014) and ASA IV (OR = 5.470, 95% CI: 1.800–16.650; *p* = 0.003) independently associated with increased odds of complications, whereas ASA II did not reach statistical significance (OR = 1.822, 95% CI: 0.930–3.570; *p* = 0.080). Operative duration remained an independent predictor (per 10 min: B = 0.070, *p* = 0.012; OR = 1.073, 95% CI: 1.015–1.134), as did advanced tumor stage (OR = 1.477, 95% CI: 1.006–2.170; *p* = 0.047) and open approach (OR = 1.682, 95% CI: 1.060–2.670; *p* = 0.028). Age, sex, BMI, and receipt of neoadjuvant therapy were not independently associated with postoperative complications (Table 6). The model demonstrated good calibration (Hosmer–Lemeshow χ2 = 5.84, *p* = 0.665) and acceptable discrimination (AUC 0.76, 95% CI: 0.70–0.82).

Univariable and multivariable logistic regression analyses of factors associated with mortality are presented in Table 7. In univariable analysis, higher EFS score was associated with increased odds of mortality (B = 0.330, *p* = 0.003; OR = 1.391, 95% CI: 1.120–1.726). Age was also significant in univariable analysis (B = 0.055, *p* = 0.018; OR = 1.057, 95% CI: 1.010–1.106). ASA physical status was strongly associated with mortality (overall *p* < 0.001), with increased odds for ASA III (OR = 4.710, 95% CI: 1.510–14.690; *p* = 0.008) and ASA IV (OR = 9.970, 95% CI: 3.060–32.470; *p* < 0.001), while ASA II did not reach statistical significance (OR = 2.225, 95% CI: 0.900–5.500; *p* = 0.080), compared with ASA I. Longer operative duration (per 10 min) was associated with mortality (B = 0.095, *p* = 0.028; OR = 1.100, 95% CI: 1.010–1.198). Advanced tumor stage (III–IV vs 0–II) (B = 0.620, *p* = 0.022; OR = 1.859, 95% CI: 1.090–3.170) and open surgical approach (vs laparoscopic) (B = 0.780, *p* = 0.010; OR = 2.180, 95% CI: 1.210–3.930) were also significant in univariable models. Sex, BMI, and receipt of neoadjuvant therapy were not significant in univariable analysis.

**Table 7:** Univariate and multivariate analyses of risk factors for mortality

Variable	Univariate B	p-value	Exp(B)	95% CI for Exp(B)	Multivariate B	p-value	Exp(B)	95% CI for Exp(B)
Edmonton score	0.330	**0.003**	1.391	1.120–1.726	0.280	**0.014**	1.323	1.060–1.652
Age (years)	0.055	**0.018**	1.057	1.010–1.106	0.020	0.390	1.020	0.975–1.067
Gender (male)	0.200	0.440	1.221	0.740–2.015	0.090	0.710	1.094	0.660–1.812
BMI	−0.035	0.580	0.966	0.855–1.092	−0.020	0.780	0.980	0.860–1.116
ASA (ref: I)		< **0.001**				**0.002**		
ASA II	0.800	0.080	2.225	0.900–5.500	0.650	0.130	1.915	0.830–4.420
ASA III	1.550	**0.008**	4.710	1.510–14.690	1.300	**0.018**	3.670	1.250–10.790
ASA IV	2.300	< **0.001**	9.970	3.060–32.470	2.000	**0.003**	7.390	2.030–26.900
Surgery duration (per 10 min)	0.095	**0.028**	1.100	1.010–1.198	0.080	**0.041**	1.083	1.003–1.169
Tumor stage (III–IV vs 0–II)	0.620	**0.022**	1.859	1.090–3.170	0.470	**0.048**	1.600	1.004–2.550
Surgical approach (Open vs Lap [ref])	0.780	**0.010**	2.180	1.210–3.930	0.650	**0.031**	1.915	1.060–3.460
Neoadjuvant treatment (Any vs None [ref])	0.210	0.430	1.234	0.740–2.050	0.100	0.720	1.105	0.650–1.880

Model fit: Model fit: Hosmer–Lemeshow test: χ^2^ = 5.12, *p* = 0.744; AUC (95% CI): 0.82 (0.75–0.89)

*Abbreviations*: *CI* confidence interval, *BMI* body mass index, *ASA* American Society of Anesthesiologists physical status

Bold values indicate statistical significance (*p* < 0.05)

In the multivariable model, EFS remained independently associated with mortality (B = 0.280, *p* = 0.014; OR = 1.323, 95% CI: 1.060–1.652). ASA physical status remained significant overall (*p* = 0.002), with ASA III (OR = 3.670, 95% CI: 1.250–10.790; *p* = 0.018) and ASA IV (OR = 7.390, 95% CI: 2.030–26.900; *p* = 0.003) independently associated with higher odds of mortality. Operative duration also remained significant (per 10 min: B = 0.080, *p* = 0.041; OR = 1.083, 95% CI: 1.003–1.169), as did advanced tumor stage (OR = 1.600, 95% CI: 1.004–2.550; *p* = 0.048) and open surgical approach (OR = 1.915, 95% CI: 1.060–3.460; *p* = 0.031). After adjustment, age was no longer independently associated with mortality (*p* = 0.390). The model showed good calibration (Hosmer–Lemeshow χ^2^ = 5.12, *p* = 0.744) and good discrimination (AUC 0.82, 95% CI: 0.75–0.89) (Table 7).

## Discussion

This prospective observational study demonstrates that preoperative frailty assessment using the Edmonton Frail Scale (EFS) provides independent and clinically relevant prognostic information for 30-day postoperative morbidity and mortality in older adults undergoing major colorectal cancer surgery. Our findings indicate that frailty cannot be adequately explained by chronological age or simple anthropometric measures such as body mass index; rather, it reflects a multidimensional clinical construct encompassing cumulative impairments in physiological, functional, and psychosocial reserve, all of which have meaningful implications for surgical outcomes. In this cohort, the EFS remained independently associated with postoperative complications and short-term mortality after adjustment for established perioperative and oncologic risk factors, including ASA physical status, tumor stage, and surgical approach. Taken together, these findings support the integration of systematic frailty assessment as a complementary component of conventional risk stratification strategies in geriatric colorectal cancer surgery.

The absence of significant differences in sex distribution (*p* = 0.183) and BMI (*p* = 0.336) across frailty strata in our cohort supports the view that frailty is a distinct multidimensional phenotype rather than a sex-dependent or purely nutritional construct [14]. While median age increased significantly with higher frailty levels (*p* = 0.028), consistent with age-related biological vulnerability [10, 14], our findings suggest that frailty captures a broader spectrum of physiological decline, including cognition, social support, and functional performance, that cannot be adequately represented by chronological age alone [15, 16]. Notably, tumor stage differed across frailty groups, underscoring the importance of adjusting for oncologic severity when interpreting postoperative risk. Importantly, EFS remained independently associated with 30-day complications and mortality in multivariable analyses after accounting for tumor stage, surgical approach, neoadjuvant treatment, and other perioperative factors. Although neoadjuvant treatment was included as a clinically relevant confounder in the multivariable models, it was not independently associated with 30-day postoperative morbidity or mortality in our cohort. Previous evidence from elective colorectal surgery indicates that frailty-based assessments help identify vulnerable older patients at increased risk for severe postoperative morbidity [17, 18]. By incorporating the Clavien–Dindo classification and standardized outcome definitions, our study further clarifies this relationship, demonstrating that higher EFS scores are associated not only with minor events but also with major (Grade III–V) complications and 30-day mortality.

In our cohort, higher ASA physical status tended to parallel increasing frailty, and both measures provided clinically meaningful signals for perioperative risk. Multivariable analysis confirmed that ASA class was independently associated with complication risk, with significantly higher odds observed in patients with ASA III–IV status. Importantly, the EFS score remained a robust independent predictor of both 30-day complications (OR 1.284; 95% CI 1.075–1.534; *p* = 0.006) and 30-day mortality (OR 1.323; 95% CI 1.059–1.653; *p* = 0.014) even after adjusting for ASA class, tumor stage, and surgical approach. This suggests that frailty reflects a broader patient vulnerability profile that extends beyond comorbidity burden to encompass cognitive, functional, and psychosocial dimensions [19, 20]. From a clinical perspective, integrating the EFS into routine preoperative assessment offers a valuable approach for individualizing surgical strategies, formulating perioperative optimization plans, and strengthening shared decision-making through more accurate patient–family counseling.

American College of Surgeons–based risk assessment approaches, such as those derived from ACS NSQIP, primarily estimate postoperative risk by integrating procedure-related factors and major comorbid conditions [13]. While these tools are valuable for quantifying surgical complexity and disease burden, they place comparatively less emphasis on domains such as functional reserve, cognition, and social vulnerability, which are particularly relevant in older adults. In this context, frailty assessment may provide complementary information to traditional ACS-based risk stratification and may help refine perioperative decision-making in geriatric surgical populations [21].

In our cohort, the Edmonton Frail Scale identified high-risk patients independently of traditional perioperative risk indicators, suggesting that frailty captures dimensions of physiological vulnerability that extend beyond those typically incorporated into ACS-based risk stratification. This conceptual distinction may explain why frailty has emerged as a strong predictor of 30-day postoperative outcomes in geriatric surgery and supports its integration alongside established ACS risk assessment frameworks to enhance preoperative risk evaluation [20, 22]. By addressing domains such as functional performance and social support, the EFS provides a more holistic view of the older patient’s resilience, which is essential for predicting recovery trajectories after major oncologic resections.

In our study, the associations between intraoperative process indicators and frailty are clinically meaningful. As frailty severity increased, operative duration lengthened (*p* = 0.027) and intraoperative blood loss rose (*p* = 0.036), suggesting that frail patients may be more susceptible to physiological stress and homeostatic perturbations during major surgery [18]. Additionally, the significant relationship between frailty and intraoperative vasopressor requirements (*p* = 0.032), along with the identification of operative duration as an independent risk factor in our multivariable model, indicates that the physiological strain of prolonged and hemodynamically challenging procedures is particularly pronounced in the frail phenotype. These findings align with reports that longer and more complex operations can increase the complication risk in frail patients through fluid imbalance, hypothermia, and an exaggerated inflammatory response [22]. From a practical standpoint, surgical–anesthetic strategies aimed at minimizing operative duration, meticulous temperature and fluid management, and close hemodynamic monitoring may be essential components of risk reduction in this vulnerable population [20].

Using a 30-day follow-up period, we observed a clear increase in postoperative medical morbidity with rising frailty severity, with the highest complication rate observed in the severely frail group (45%; Table 4). Importantly, when complication severity was classified according to the Clavien–Dindo system, frailty was associated not only with minor events (Grade I–II) but also with major complications (Grade III–V) and 30-day mortality (Table 4). These findings underscore that frailty is clinically relevant for identifying patients at risk for both mild and severe postoperative adverse events following major colorectal cancer surgery [17, 18]. Consistent with our multivariable analyses, which demonstrated an independent association between EFS and both 30-day complications and mortality, this graded relationship supports the role of structured frailty assessment as an important adjunct to conventional perioperative risk evaluation in geriatric surgical patients [19, 22]. Furthermore, our results align with prior studies using the EFS in acute care settings, which have consistently shown that higher frailty scores are predictive of increased postoperative morbidity and mortality [8, 23, 24].

In our analyses, higher EFS scores were associated with longer hospital length of stay and a higher burden of postoperative adverse outcomes, supporting the clinical relevance of frailty as a multidimensional marker of reduced physiological reserve in older surgical patients [15, 18]. Correlation analyses further supported these associations, demonstrating positive relationships between EFS scores and hospital length of stay, postoperative complications, and 30-day mortality. Importantly, EFS demonstrated excellent discrimination for 30-day postoperative complications on ROC analysis (AUC 0.928; 95% CI 0.886–0.970), with an optimal cut-off of approximately 6.5, yielding high sensitivity (96.2%) and good specificity (76.9%). These findings suggest that EFS may be a pragmatic tool for perioperative triage, risk communication, and guiding the intensity of perioperative monitoring and optimization strategies in geriatric colorectal cancer surgery. Nevertheless, potential overestimation related to the single-center design and the limited number of outcome events cannot be fully excluded and should be considered when interpreting these findings.

Given that AUC values reported for the EFS in some surgical literature are often moderate, ranging approximately from 0.67 to 0.69 [23, 24], the excellent discrimination observed in our cohort (AUC 0.928) warrants careful interpretation. This high performance may be attributable to several factors, including the standardization of EFS measurement in a prospective setting, the specific focus on a high-risk geriatric population undergoing major colorectal cancer surgery, and the use of standardized 30-day outcome definitions [25]. Furthermore, the inclusion of both medical and surgical complications within a 30-day window, graded by the Clavien–Dindo system, may have enhanced the scale's ability to capture the full spectrum of frailty-related vulnerability. These observations suggest that the EFS exhibits context-sensitive performance and may emerge as a particularly powerful scoring tool in high-stakes oncogeriatric surgery, where physiological reserve is a primary determinant of recovery [6, 20].

When examining the spectrum of 30-day adverse events, pulmonary complications emerged as the most frequent medical morbidity in our cohort, followed by cardiac events and delirium. Pulmonary complications, surgical site infections, and postoperative ileus constituted the most frequent postoperative medical complications, with a clear clustering in patients classified as severely frail. This pattern is consistent with reports across various surgical domains indicating that pulmonary complications constitute a dominant component of postoperative morbidity in geriatric populations, with a marked increase in incidence as frailty worsens [20, 23]. Notably, the observation that delirium occurred predominantly in the severely frail group underscores the close relationship between frailty and neuropsychiatric vulnerability, likely mediated by reduced cognitive reserve and an exaggerated systemic inflammatory response to surgical stress. These findings support the prioritization of delirium-prevention bundles in frail patients, including preoperative cognitive screening, early postoperative mobilization, sleep hygiene, and meticulous management of pain and polypharmacy [26, 27]. By identifying these specific high-risk patterns, the EFS can guide targeted perioperative interventions to mitigate the most common medical complications in the onco-geriatric population.

In the mortality analysis, the persistence of the Edmonton Frail Scale as an independent risk factor (OR = 1.323; 95% CI: 1.060–1.652) indicates that frailty not only increases the likelihood of postoperative complications but also amplifies the overall severity of adverse outcomes. In addition to frailty, prolonged length of hospital stays and increased intraoperative blood loss were independently associated with early postoperative mortality, highlighting the cumulative impact of physiological vulnerability and perioperative stress. These findings are consistent with large-scale studies across surgical disciplines demonstrating a strong association between frailty and short-term mortality, including 30-day and 90-day outcomes [7, 26, 28].

Moreover, the absence of a statistically significant association between post-anesthesia care unit (PACU) length of stay and frailty in our cohort suggests that system-level factors, such as institutional protocols governing recovery unit admission and discharge, bed availability, and predefined thresholds for clinical stability, may play a substantial role in these decisions. In contrast, the significantly longer total hospital length of stay observed in frail patients (8.8 ± 5.6 vs. 7.1 ± 3.0 days; *p* = 0.023) indicates that frailty is associated with a slower pace of postoperative recovery and more complex discharge planning. Beyond patient-level vulnerability, institutional care pathways and postoperative practices—including the lack of a standardized enhanced recovery after surgery protocol and the requirement for stoma education prior to discharge—may have further contributed to prolonged hospitalization in this population.

Our findings have multilayered clinical implications for the perioperative management of older adults undergoing major colorectal cancer surgery. First, integrating routine preoperative EFS screening could standardize risk communication, realistically align patient and family expectations, and strengthen shared decision-making. Second, identifying patients with moderate-to-severe frailty may facilitate targeted prehabilitation—encompassing nutritional optimization, resistance training, and respiratory physiotherapy—alongside medication reconciliation and the management of anemia and sarcopenia, all of which have the potential to reduce postoperative complication risk. Intraoperatively, strategies such as individualized hemodynamic targets, judicious fluid management, and delirium-conscious anesthetic regimens are particularly salient in frail patients. In the postoperative period, early mobilization, multimodal analgesia, and structured geriatric co-management may further mitigate morbidity and support recovery. Moving forward, future directions should prioritize rigorous external validation of the EFS across diverse centers and oncologic procedures to establish context-appropriate cut-off thresholds and demonstrate real-world clinical utility. Furthermore, pragmatic, patient-centered trials are needed to evaluate the effectiveness and cost-efficiency of EFS-guided perioperative care bundles. Finally, the development of implementable risk models that integrate the EFS with biologically grounded markers—such as systemic inflammation, sarcopenia metrics, and cardiopulmonary reserve—should be evaluated head-to-head against existing scores to quantify their incremental predictive value and feasibility within routine clinical workflows.

## Limitations

This study has several limitations that warrant consideration. Although prospective in design, the research was conducted at a single tertiary oncology center, which may limit the generalizability of our findings to clinical settings with different surgical volumes or patient demographics. While we extended the follow-up period to 30 days to capture clinically significant morbidity, post-discharge events were ascertained through structured telephone interviews with patients or caregivers. Although this is a widely accepted method, it remains susceptible to recall bias and may lead to the underreporting of minor complications that did not necessitate hospital readmission. Furthermore, while our multivariable models accounted for key confounders—including tumor stage, surgical approach, and neoadjuvant treatment—more granular procedural details, such as specific anastomotic techniques or the extent of lymphadenectomy, were not captured in a standardized format. Additionally, our hospital length of stay data may reflect institutional factors, such as the absence of a formal Enhanced Recovery After Surgery (ERAS) program and the time required for stoma education, which may limit direct comparisons with centers utilizing established ERAS pathways. Finally, frailty was assessed at a single preoperative time point; however, frailty is a dynamic state that may evolve in response to perioperative optimization. Future multicenter studies incorporating longitudinal frailty assessments and standardized surgical adjudication are essential to further refine risk stratification and evaluate the impact of frailty-guided interventions on the recovery of older adults.

## Conclusion

In conclusion, our study underscores that frailty, as measured by the Edmonton Frail Scale, is a powerful and independent predictor of 30-day morbidity and mortality in older adults undergoing colorectal cancer surgery. Even after accounting for oncologic stage and surgical approach, the EFS identified a unique dimension of physiological vulnerability that conventional risk tools often overlook. These findings suggest that shifting our focus from chronological age to a multidimensional frailty assessment is not merely a statistical improvement, but a clinical necessity. Integrating the EFS into routine practice offers a more compassionate and precise pathway to individualize perioperative care, optimize patient safety, and ultimately ensure that surgical interventions align with the functional resilience of the older patient.

## Data Availability

The data supporting the findings of this study are not publicly available due to patient confidentiality and ethical restrictions. However, the data can be made available from the corresponding author upon reasonable request and with the approval of the institutional ethics committee.

## Abbreviations

AUC: Area Under the Curve
ASA: American Society of Anesthesiologists (Physical Status Classification)
BMI: Body Mass Index
CI: Confidence Interval
EFS: Edmonton Frail Scale
ICU: Intensive Care Unit (metinde dolaylı olarak bakım birimleri bağlamında)
IQR: Interquartile Range
LOS: Length of (Hospital) Stay
NA: Noradrenaline
OR: Odds Ratio
PACU: Post-Anesthesia Care Unit
ROC: Receiver Operating Characteristic
SD: Standard Deviation
